# Toxicity of nano- and ionic silver to embryonic stem cells: a comparative toxicogenomic study

**DOI:** 10.1186/s12951-017-0265-6

**Published:** 2017-04-11

**Authors:** Xiugong Gao, Vanessa D. Topping, Zachary Keltner, Robert L. Sprando, Jeffrey J. Yourick

**Affiliations:** Division of Applied Regulatory Toxicology, Office of Applied Research and Safety Assessment, Center for Food Safety and Applied Nutrition, U.S. Food and Drug Administration, 8301 Muirkirk Road, Laurel, MD 20708 USA

**Keywords:** Silver nanoparticles, Silver ion, Embryonic stem cell, Developmental toxicity, Transcriptomics

## Abstract

**Background:**

The widespread application of silver nanoparticles (AgNPs) and silver-containing products has raised public safety concerns about their adverse effects on human health and the environment. To date, in vitro toxic effects of AgNPs and ionic silver (Ag^+^) on many somatic cell types are well established. However, no studies have been conducted hitherto to evaluate their effect on cellular transcriptome in embryonic stem cells (ESCs).

**Results:**

The present study characterized transcriptomic changes induced by 5.0 µg/ml AgNPs during spontaneous differentiation of mouse ESCs, and compared them to those induced by Ag^+^ under identical conditions. After 24 h exposure, 101 differentially expressed genes (DEGs) were identified in AgNP-treated cells, whereas 400 genes responded to Ag^+^. Despite the large differences in the numbers of DEGs, functional annotation and pathway analysis of the regulated genes revealed overall similarities between AgNPs and Ag^+^. In both cases, most of the functions and pathways impacted fell into two major categories, embryonic development and metabolism. Nevertheless, a number of canonical pathways related to cancer were found for Ag^+^ but not for AgNPs. Conversely, it was noted that several members of the heat shock protein and the metallothionein families were upregulated by AgNPs but not Ag^+^, suggesting specific oxidative stress effect of AgNPs in ESCs. The effects of AgNPs on oxidative stress and downstream apoptosis were subsequently confirmed by flow cytometry analysis.

**Conclusions:**

Taken together, the results presented in the current study demonstrate that both AgNPs and Ag^+^ caused transcriptomic changes that could potentially exert an adverse effect on development. Although transcriptomic responses to AgNPs and Ag^+^ were substantially similar, AgNPs exerted specific effects on ESCs due to their nanosized particulate form.

**Electronic supplementary material:**

The online version of this article (doi:10.1186/s12951-017-0265-6) contains supplementary material, which is available to authorized users.

## Background

The use of engineered nanoscale materials in consumer products has increased dramatically over the past decade. Only 54 consumer products claimed to contain nanomaterials in 2005, but the number has surged to more than 1600 today [1]. It has been estimated that of all the nanomaterials manufactured, silver nanoparticles (AgNPs) have the highest degree of commercialization [2], owing largely to their broad spectrum of antimicrobial activities against bacteria, fungi, and viruses, including HIV and SARS [3, 4]. Currently, a large variety of consumer products contain AgNPs, including food packaging materials, dietary supplements, cosmetics, textiles, electronics, household appliances, medical devices, water disinfectants, and room sprays [5].

The widespread application of AgNPs has raised public safety concerns about their adverse effects on human health and the environment. Data from in vitro studies demonstrated that AgNPs induce cytotoxicity and genotoxicity through the production of reactive oxygen species (ROS), DNA damage, cell cycle arrest, ultimately leading to inflammation, apoptosis, and cell death [6, 7]. In addition, in vivo studies [8–10] demonstrated that AgNPs enter the blood circulation and accumulate in organs including the brain, kidneys, lungs, spleen, testes and primarily the liver. The ability of AgNPs to enter the blood stream [10] and cross through the blood–brain barrier [11] points toward the potential of these nanoparticles to migrate into the uterus, placenta and embryo thus causing developmental toxicity [12].

Embryonic stem cells (ESCs) have been shown to faithfully recapitulate stages of early embryo development and are increasingly used as an in vitro model for developmental toxicity testing [13]. An in vitro test has been developed over two decades ago to evaluate embryotoxicity of chemical compounds using mouse embryonic stem cells (mESCs) [14]. The so-called embryonic stem cell test (EST) was latterly validated by the European Committee for the Validation of Alternative Methods (ECVAM) [15] and is currently used in the pharmaceutical industry [16]. In addition, implementing toxicogenomics into the EST improves its application domain and predictability [17], and has been shown as a promising alternative method for developmental toxicity testing [18]. Despite these progresses, only a few studies reported the toxicity of AgNPs in ESCs [19–21]. Moreover, little is known about the mechanisms of AgNP toxicity in ESCs at the molecular level.

At present, there is no consensus on the mechanisms of action for AgNP cytotoxicity and research findings are equivocal. For example, Bouwmeester et al. [22] found in an in vitro intestinal epithelium coculture model that treatment with AgNPs induced regulation of the same set of genes as with silver nitrate (AgNO_3_), and 6–17% of the silver content in the AgNPs suspensions was found in the ionic form (Ag^+^). The authors therefore speculated that the observed gene regulation was exerted by Ag^+^ released from the AgNPs. On the other hand, other studies suggest that AgNP toxicity is related to Ag^+^ and to the nanosize as well [23, 24]. Thus, AgNP cytotoxicity is a complex phenomenon and more research is needed to distinguish cellular effects triggered by the nanosized particle from those by Ag^+^ released from the nanoparticles.

The present work aimed at unraveling the molecular mechanisms of AgNP toxicity in ESCs. Using microarrays, we characterized transcriptomic changes induced by citrate-coated 20 nm AgNPs during spontaneous differentiation of a C57BL/6-derived mESC cell line. This cell line has been demonstrated to detect global gene expression changes induced by a variety of developmental toxicants [18]. To our knowledge, no toxicogenomic study on AgNPs has been reported hitherto in ESCs. We also compared the gene expression changes to those induced by Ag^+^ (silver acetate). We evaluated the potential toxicity of AgNPs with emphasis on oxidative stress and apoptosis by correlating cellular responses to gene expression patterns, which provides a mechanistic understanding of the toxicity of AgNPs in ESCs.

## Methods

### Materials

BioPure 20 nm AgNP citrate solutions, at AgNP concentration of 1.0 mg/ml, were purchased from nanoComposix (San Diego, CA). The AgNPs were extensively washed with the suspending solvent to remove residual reactants from the manufacturing process, and were sterile filtered and tested for endotoxin contamination before delivery. The stock solution as obtained from the manufacturer was stored at 4 °C in the dark throughout the study, and was diluted to the designated concentrations using medium or water and vortexed briefly (2500 rpm, 5 s.) before the characterization or the exposure experiments. ReagentPlus grade silver acetate was purchased from Sigma-Aldrich (St. Louis, MO), and solutions of 1.00 mg/ml silver ion (Ag^+^) equivalent to 1.53 mg/ml silver acetate were prepared fresh before exposure. All other chemicals used in this study were of molecular biology grade and were obtained from Sigma-Aldrich unless otherwise stated.

### AgNP characterization

The AgNPs were characterized using dynamic light scattering (DLS) and transmission electron microscopy (TEM) to confirm the nanoparticle size distribution reported by the manufacturer. To measure the hydrodynamic diameters, samples of AgNPs were suspended at 50 µg/ml in deionized (DI) water or in culture medium, immediately placed in a disposable cuvette, and analyzed at 25 °C for 2 min per run on a Brookhaven 90Plus/BI-MAS Particle Size Analyzer (Holtsville, NY). Each sample was run in triplicate, with the polydispersity index (PDI) results presented as an average of the three measurements.

To characterize AgNP structure, shape and size uniformity, TEM was performed at an accelerating voltage of 80 kV on a JEOL JEM-1011 transmission electron microscope (Peabody, MA) equipped with a bottom-mounted Gatan Orius SC1000A camera and a Gatan Microscopy Suite software platform (Pleasanton, CA). Grids were prepared by placing a 10 μl drop of AgNP sample solution (50 µg/ml) onto a formvar/carbon-coated copper 100 mesh grid.

The stability of the AgNP suspension was monitored at several time points during a 24 h incubation period by dilution in culture media to 5.0 μg/ml followed by Ultraviolet–visible (UV–vis) spectroscopy using a SpectraMax i3 spectrometer from Molecular Devices (Sunnyvale, CA).

### Silver concentration assessment using ICP-MS

Mass concentration of silver was determined with inductively coupled plasma mass spectroscopy (ICP-MS) on a 7700 series ICP-MS from Agilent Technologies (Santa Clara, CA) equipped with on-line internal standard delivery. Total silver was analyzed using *m/z* 107 and Y and In as internal standards. Calibration standards were prepared by dilution from a 1000 ppm silver standard from Inorganic Ventures (Christiansburg, VA). A calibration curve was verified for each analysis using dilutions from a 1 ppm silver standard from SPEX CertiPrep (Metuchen, NJ). To assess silver concentration in the nanoparticle suspensions, tubes were sonicated while an aliquot for dilution was taken out and acidified with 800 µl of concentrated nitric acid. The samples were then diluted to 10 ml with a 4% HNO_3_ 0.5% HCl solution. For analysis of the supernatants, AgNP suspensions were subjected to centrifugation at 25,000×*g* for 90 min, using a WX Ultra Series centrifuge with a F50L-24 × 1.5 rotor (Thermo Scientific). Supernatants were carefully separated from pellets and silver concentration assessed.

### Pluripotent mouse embryonic stem cell culture

Pluripotent ESGRO complete adapted C57BL/6 mESCs, which have been pre-adapted to serum-free and feeder-free culture condition, were obtained from EMD Millipore (Billerica, MA) at passage 12 (with 80% normal male mouse karyotype). The cells were seeded in cell culture flasks (Nunc, Roskilde, Denmark) coated with 0.1% gelatin solution (EMD Millipore), and maintained at 37 °C in a 5% CO_2_ humidified incubator at standard densities (i.e., between 5 × 10^4^/cm^2^ and 5 × 10^5^/cm^2^) in ESGRO Complete Plus Clonal Grade Medium (EMD Millipore). The medium contains leukemia inhibitory factor (LIF), bone morphogenic protein 4 (BMP-4), and a glycogen synthase kinase-3b inhibitor (GSK3b-I) to help maintain pluripotency and self-renewal of the ESCs. Cells were passaged every 2–3 days (when reaching 60% confluence) with ESGRO Complete Accutase (EMD Millipore) at about 1:6 ratio. C57BL/6 mESCs maintain a stable karyotype under the above passaging condition. The cells used in the current study were at passage 18.

### Cell differentiation through embryoid body formation

Induction of differentiation was achieved through embryoid body (EB) formation in hanging drop culture following a procedure adapted from De Smedt et al. [25]. In brief, stem cell suspensions were prepared on ice at a concentration of 3.75 × 10^4^ cells/ml in ESGRO Complete Basal Medium (EMD Millipore), which does not contain LIP, BMP-4, or GSK3b-I. About 50 drops (each of 20 µl) of the cell suspension were placed onto the inner side of the lid of a 10-cm Petri dish filled with 5 ml phosphate buffered saline (PBS) (EMD Millipore) and incubated at 37 °C and 5% CO_2_ in a humidified atmosphere. After 3 days, EBs formed in the hanging drops were subsequently transferred into 6-cm bacteriological Petri dishes (Becton–Dickinson Labware, Franklin Lakes, NJ) and were exposed to AgNPs or Ag^+^. The EBs had an average diameter of 330–350 μm.

### Cytotoxicity assay

Cytotoxicity was measured both in adherent (monolayer) culture and in EB culture by MTS assay using the CellTiter 96 AQueous One Solution Cell Proliferation Assay kit from Promega (Madison, WI), following instructions from the manufacturer. For adherent culture, pluripotent C57BL/6 mESC colonies cultured in ESGRO Complete Plus Clonal Grade Medium were dissociated with ESGRO Complete Accutase and a single-cell suspension at 1.0 × 10^5^ cells/ml was prepared in ESGRO Complete Basal Medium. The cells were seeded in 96-well cell culture grade flat bottom plates (Nunc) coated with 0.1% gelatin (EMD Millipore) at 100 µl/well (1.0 × 10^4^ cells/well) and allowed to adhere overnight at 37 °C with 5% CO_2_. After 24 h, 100 µl medium containing 2× final concentrations of AgNPs or Ag^+^ (0.1–50 µg/ml) was added to the test wells. In control wells, the same volume of medium was added as a vehicle control. The treatment was maintained for 24 h. At the end of the exposure, 20 μl of CellTiter 96 AQueous One Solution Cell Proliferation Assay reagent was added to each well that contained 100 μl medium. After 3 h incubation at 37 °C, the resultant absorbance was recorded at 490 nm using a SpectraMax i3 plate reader (Molecular Devices). Each concentration was tested in sextuplicate and repeated six times. To correct for interference of AgNPs or Ag^+^ on MTS assay, a parallel control plate was set up with identical concentrations of AgNPs or Ag^+^ but without seeded cells. The control plate was treated otherwise the same way as the test plate. The readings of the control plate were then subtracted from the corresponding wells of the test plate, and the resultant values were used in the dose–response plot.

For cytotoxicity assay in EB state, hanging drops were set up as described above. After 3 days, EBs were subsequently transferred into 6-cm bacteriological Petri dishes (Becton–Dickinson Labware) and treated with designated concentrations of AgNPs or Ag^+^ for 24 h. Afterwards, 50 EBs were harvested per compound concentration. The EBs were allowed to precipitate and supernatant was removed, and were subsequently dissociated using ESGRO Complete Accutase (EMD Millipore). Cells were then resuspended in 700 μl ESGRO Complete Basal Medium (EMD Millipore), and 100 μl of the single cell suspension was pipetted into each well (in sextuplicate) of 96-well cell culture grade flat bottom plates (Nunc). Subsequent MTS assay was the same as for the adherent culture described above. The experiment was repeated independently three times.

### AgNP/Ag^+^ exposure and RNA isolation

EB differentiation cultures at day 3 were exposed to the desired concentrations of AgNPs or Ag^+^ for 24 h. EBs were collected after exposure. Three biological replicates were used for each condition. Treatment with AgNPs or Ag^+^ at the concentrations used did not affect EB sizes (data not shown). EBs were lysed in RLT buffer (Qiagen; Valencia, CA) supplemented with β-mercaptoethanol, homogenized by QIAshredder (Qiagen), and kept in a −80 °C freezer until further processing. Total RNA was isolated on the EZ1 Advanced XL (Qiagen) automated RNA purification instrument using the EZ1 RNA Cell Mini Kit (Qiagen) following the manufacturer’s protocol, including an on-column DNase digestion. RNA concentration and purity (260/280 ratio) were measured with a NanoDrop 2000 UV–Vis spectrophotometer (NanoDrop Products, Wilmington, DE). Integrity of RNA samples was assessed by an Agilent 2100 Bioanalyzer (Santa Clara, CA) with the RNA 6000 Nano Reagent Kit from the same manufacturer.

### RNA processing and microarray experiment

The total RNA samples were preprocessed for hybridization to Mouse Gene 2.0 ST Array (Affymetrix, Santa Clara, CA) using the GeneChip WT PLUS Reagent Kit (Affymetrix) following the manufacturer’s protocol. For each sample, 100 ng of total RNA was used. Subsequent hybridization, wash, and staining were carried out using the Affymetrix GeneChip Hybridization, Wash, and Stain Kit and the manufacturer’s protocols were followed. The chips were then scanned on Affymetrix GeneChip Scanner 3000 7G and the image (DAT) files were preprocessed using the Affymetrix GeneChip Command Console (AGCC) software v.4.0 to generate cell intensity (CEL) files. Prior to data analysis, all arrays referred to in this study were assessed for data quality using the Affymetrix Expression Console software v.1.3 and all quality assessment metrics (including spike-in controls during target preparation and hybridization) were found within boundaries. The data set has been deposited in Gene Expression Omnibus (GEO; http://www.ncbi.nlm.nih.gov/geo/) of the National Center for Biotechnology Information with accession number GSE79766.

### Microarray data processing and analysis

The values of individual probes belonging to one probe set in CEL files were summarized using the robust multi-array average (RMA) algorithm [26] embedded in the Expression Console software v.1.3 (Affymetrix), which comprises of convolution background correction, quantile normalization, and median polish summarization. Normalized data for all samples were then analyzed by unsupervised principal component analysis (PCA) [27] and hierarchical cluster analysis (HCA) [28], using the U.S. FDA’s ArrayTrack software system [29, 30], to identify patterns in the dataset and highlight similarities and differences among the samples. Subsequently, differentially expressed genes (DEGs) were selected using one-way analysis of variance (ANOVA) using the Affymetrix Transcriptome Analysis Console (TAC) software v.2.0. Prior to making comparisons, a gene filtering procedure was applied to exclude probesets that appeared to be unexpressed in all sample groups. For each comparison between two experimental groups, the fold change (FC) of every annotated gene, together with their corresponding *p* value, was used for selection of DEGs with cutoff values indicated in the text.

### Gene ontology and pathway analysis

The DEGs were subjected to gene ontology (GO) analysis using the Database for Annotation, Visualization, and Integrated Discovery (DAVID) [31, 32] to find overrepresentations of GO terms in the biological process (BP) category (GOTERM_BP_FAT). As background, the *Mus musculus* (mouse) whole genome was used. Statistical enrichment was determined using default settings in DAVID. The statistically enriched GO terms were classified using the web tool CateGOrizer [33] based on GO Slim. Pathway analysis was conducted with the Ingenuity Pathway Analysis (IPA) software using default settings to identify canonical pathways and pathway interaction networks associated with the DEGs.

### Measurement of oxidative stress and apoptosis by flow cytometric analysis

EBs were exposed to 5.0 µg/ml of AgNPs or Ag^+^ the same way as in the gene expression study described above. As a positive control for both oxidative stress and apoptosis, EBs were also treated with 1 µM staurosporine. After treatment, EBs were collected and dissociated into single cells using the Embryoid Body Dissociation Kit from Miltenyi Biotec (San Diego, CA) following instruction from the manufacturer. Subsequent flow cytometry measurements of oxidative stress and apoptosis were conducted on a Guava easyCyte 8HT Flow Cytometer from EMD Millipore with a kit from the same manufacturer. The MitoStress Kit allows for the simultaneous measurement of mitochondrial superoxide generation, detected by membrane permeant dye MitoSox Red, and phosphatidyl serine expression on the cell surface of apoptotic cells as assessed by Annexin V binding. Data collection was performed using the InCyte and the CellCycle programs, both included in the guavaSoft software suite (ver. 3.1.1), and instructions from the manufacturer were followed.

### Statistical analysis

Statistical analysis for data other than the microarray data was performed in KaleidaGraph v.4.03 of Synergy Software (Reading, PA) using one way ANOVA followed by Tukey HSD post hoc test.

## Results

### Silver nanoparticle characterization

**Fig. 1:**
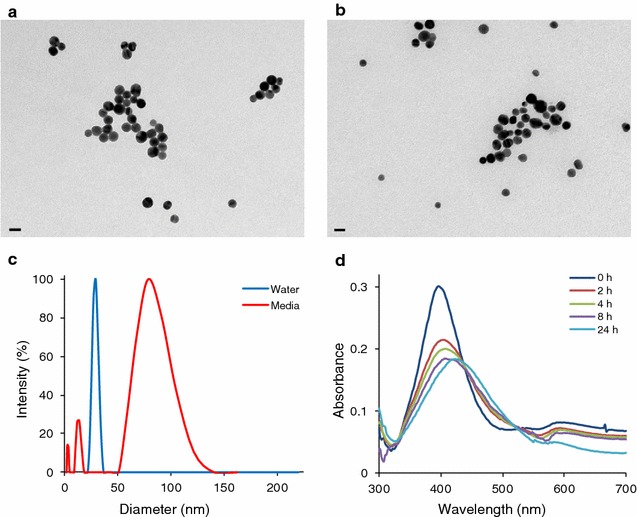
Characterization of AgNPs. **a** Typical TEM images of AgNPs in water. **b** Typical TEM images of AgNPs in cell culture medium. The *size bar* at the *bottom left corner* of the images represents 20 nm. **c** Comparison of hydrodynamic size distribution of AgNPs in water and in cell culture medium. **d** Monitoring of AgNP stability by UV–vis spectra over 24 h incubation in cell culture medium

The physicochemical properties of the 20 nm AgNPs are summarized in Table 1. The values in water agree well with those provided by the manufacturer. TEM analysis indicated that the AgNPs were spherical in shape and uniform in size with an average diameter of 20.4 ± 3.2 nm in water (Fig. 1a) and of 20.2 ± 4.1 nm in medium (Fig. 1b). The particle size distribution was narrow, with few particles >30 or <10 nm present. As is typical for nanomaterials, the hydrodynamic diameter of the AgNPs in water measured by DLS was found to be slightly larger (29.3 nm) than the physical diameter measured by TEM (20.4 nm). The hydrodynamic diameter increased substantially in the medium (78.6 nm) (Fig. 1c), probably due to slight agglomeration. Two small peaks appeared in the low diameter range on the DLS plot, probably resulted from protein species found in the media. The AgNPs had a low PDI (0.048) in water but much higher (0.349) in medium, indicating good stability in water but not in the culture medium. UV–Vis analysis in media (Fig. 1d) revealed colloidal homogeneity at the beginning (0 h) as reflected in the surface plasmon resonance with a characteristic peak ~400 nm. However, the peak intensity decreased rapidly within the first 2 h and then slowly between 2 and 8 h, with the peak broadened and shifted towards higher wavelengths, suggesting agglomeration of the particles in the medium. After 8 h, no further decrease in peak intensity was observed, although the peak position further shifted from 408 nm (8 h) to 424 nm (24 h).

**Table 1:** Physicochemical properties of silver nanoparticles

Dispersant	Concentration (mg/ml)	TEM diameter (nm)	DLS diameter (nm)	PDI	ζ-potential (mV)
2 mM citrate^a^	1.06	20.6 ± 3.6	26.2	–	−39.8
Water	1.01	20.4 ± 3.2	29.3	0.048	–
Medium^b^	–	20.2 ± 4.1	78.6	0.349	–

*TEM* transmission electron microscopy, *DLS* dynamic light scattering; *PDI* polydispersity index

^a^Values provided by the manufacturer for the lot used in the current study

^b^ESGRO Complete Basal Medium (EMD Millipore) used for EB formation and exposure

– Data not available

To determine the amount of Ag^+^ released from AgNPs, AgNPs were soaked in the medium at various concentrations (1.0, 2.0, 5.0 and 10.0 µg/ml) at 37 °C. After 24 h, AgNPs were removed from the medium by ultracentrifugation, and Ag^+^ in the supernatant was measured by ICP-MS (Additional file 1: Figure S1). At 5.0 µg/ml, the initial silver ion (Ag^+^) fraction of the AgNP solution was 1.82% (0.091 µg/ml) but increased to 21.46% (1.073 µg/ml) after 24 h incubation at 37 °C.

### Cytotoxicity of silver nanoparticles and silver ion on mouse embryonic stem cells

**Fig. 2:**
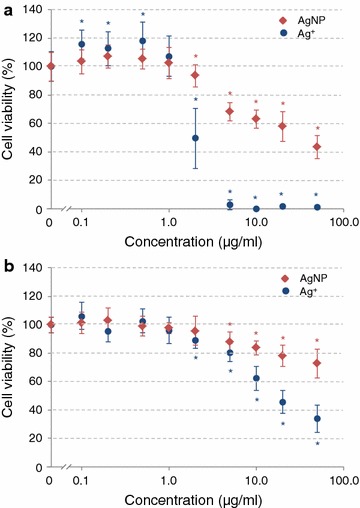
Dose response of AgNP and Ag^+^ exposure. Differentiating mESCs in adherent culture (**a**) or in EB state (**b**) were exposed to different concentrations of AgNPs and Ag^+^ for 24 h, and cell viability was measured by MTS assay. The data were normalized to the control (100%) and expressed as mean ± SD. *p < 0.05

Differentiating mESCs, either in adherent culture or in EB culture, were exposed to varying concentrations of AgNPs or Ag^+^ for 24 h, and cell viability was measured by MTS assay (Fig. 2). In adherent culture, both Ag^+^ and AgNPs exhibited a significant concentration-dependent cytotoxicity at concentrations >1.0 μg/ml (Fig. 2a). However, Ag^+^ appeared much more potent than AgNPs, causing nearly complete cell death at as low as 5.0 μg/ml. In comparison, >40% cells survived after exposing to 50 μg/ml of AgNPs. Interestingly, low concentrations of Ag^+^ (up to 0.5 μg/ml) increased cell viability almost 20% relative to the unexposed control, suggesting accelerated mESC proliferation and/or differentiation. Similar stimulating effects were also observed for AgNPs, albeit the effects were not statistically significant.

In EB state, differentiating mESCs appeared more resistant to AgNP and Ag^+^ cytotoxicity. Ag^+^ did not cause significant cytotoxicity at concentrations <2.0 μg/ml, nor did AgNPs at concentrations <5.0 μg/ml. Concentration-dependent decrease of cell viability at higher concentrations was also less severe than in adherent culture for both AgNPs and Ag^+^. At 50 µg/ml, >70% cells remained alive after exposure to AgNPs, and ~35% cells survived Ag^+^ exposure.

The morphological changes of the differentiating EBs (Additional file 2: Figure S2) corroborated the dose–response data shown above. After the 3 day EB formation period and prior to exposure, the mESCs formed compact three-dimensional aggregates in spheroid shape, with a diameter of about 330–350 µm. During the following 24 h exposure period, the cells on the surface outgrew and formed a loose outer shell of 2–3 cells thick around the compact “core”. In control samples and those of lower concentration exposure, the outer shell was intact with a smooth contour. However, when AgNPs exceeded 5.0 µg/ml, cells on the outer shell started to fall off. At 10 µg/ml and above, almost all cells on the outer shell fell off, whereas the core of the EBs was largely intact. In the case of Ag^+^ exposure, cells stared to fall off the shell at 2.0 µg/ml. At 10 µg/ml and above, not only the outer shell completely disappeared, but the size of the core also decreased significantly.

The use of 5.0 μg/ml as the exposure concentration for AgNPs in the following microarray experiments was based on the cytotoxicity data described above. At this concentration, ~10% cell death (EC10) was observed for AgNPs in the EBs, as measured by MTS assay (Fig. 2b). Two concentrations of Ag^+^ were used for comparison: 1.0 and 5.0 μg/ml. The lower concentration (1.0 μg/ml) corresponds to the concentration of Ag^+^ released from 5.0 μg/ml AgNPs into medium after 24 h incubation. The higher concentration (5.0 μg/ml) matches the total silver mass. Cell death in EBs after 24 h incubation at these concentrations of Ag^+^ was 4 and 20%, respectively (Fig. 2b).

### Global gene expression profiling

**Fig. 3:**
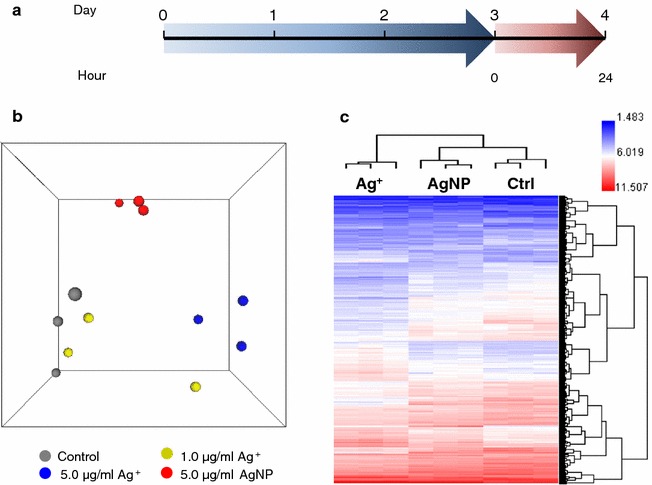
Global gene expression responses of AgNPs and Ag^+^ in differentiating mESCs. **a** Schematic representation of the experimental procedure. The *slate blue arrow* covers the embryoid body (EB) formation stage. Hanging drops were set up on day 0 and EBs formed on day 3. ESC differentiation started from day 3 onwards. AgNP or Ag^+^ exposure is shown by the *maroon arrow*, which lasted for 24 h (from day 3 to day 4). **b** Principal component analysis (PCA) using 16,510 filtered genes to cluster samples based on their similarities or dissimilarities. The three axes represent the first three principal components identified by the analysis. **c** Hierarchical cluster analysis using 459 unique DEGs to cluster samples based on their similarities or dissimilarities. The clustering was performed through Ward’s minimum variance linkage on normalized expression data which are in log2 scale and color coded as shown in the scheme at the *top right corner*. The *tree* on the *right of the image* shows clusters of genes (names not shown), while the *tree* on the *top of the image* shows clusters of samples. Samples labeled as Ag^+^ were treated with 5.0 µg/ml of Ag^+^. Those treated with 1.0 µg/ml Ag^+^ were omitted from the analysis

Cells in differentiating EBs were exposed for 24 h to 5.0 µg/ml of AgNPs, or to 1.0 or 5.0 µg/ml Ag^+^ ions (Fig. 3a), and global gene expression changes were profiled using Affymetrix Mouse Gene 2.0 ST Array. Principal component analysis (PCA) of the microarray data showed that the biological triplicates of each treatment group (control, AgNP, and Ag^+^) clustered roughly together and separated from one another except for the replicates of 1.0 µg/ml Ag^+^ (Fig. 3b). Two of the replicates fell in the same area with the controls, but the remaining one fell close to the 5.0 µg/ml Ag^+^ samples, far away from the other two. Due to the divergence between the replicates of the 1.0 µg/ml Ag^+^ treatment group, they were excluded from further analysis. For the remainder of this report, treatment with 5.0 µg/ml Ag^+^ will be simply referred to as treatment with Ag^+^. Hierarchical clustering analysis (HCA) clustered the rest of the triplicates into respective treatment groups (Fig. 3c). In addition, the HCA indicated that the gene expression pattern of the samples treated with AgNPs was more related to that of the controls than to that of the Ag^+^-treated samples.

The number of differentially expressed genes (DEGs) was considerably lower in cells exposed to AgNPs than in cells exposed to Ag^+^ (Table 2). Using a fold change (FC) cutoff value of |FC| ≥ 1.5 and *p* < 0.05, 101 DEGs were identified in AgNP-treated cells (Additional file 3: Table S1), and 400 DEGs in Ag^+^-treated cells (Additional file 4: Table S2). Among these genes, only 17 and 133 had |FC| ≥ 2.0 for AgNP- and Ag^+^-treated cells respectively, indicating that in both cases the majority of the DEGs had a |FC| between 1.5 and 2.0. For both AgNP- and Ag^+^-treated cells, the number of upregulated genes was smaller than that of downregulated genes. However, when directly comparing AgNP-treated cells to Ag^+^-treated cells, the number of upregulated genes was higher than that of downregulated genes. There were 173 DEGs identified between AgNP-treated and Ag^+^-treated cells (Additional file 5: Table S3).

**Table 2:** Number of differentially expressed genes (|FC| ≥ 1.5, *p* < 0.05)

	All	Upregulated	Downregulated
AgNP vs Ctrl	101 (17)^a^	43 (6)	58 (11)
Ag^+^ vs Ctrl	400 (133)	137 (41)	263 (92)
AgNP vs Ag^+^	173 (39)	116 (32)	57 (7)

^a^Values in the parentheses indicate the number of genes that had |FC| ≥ 2.0

**Fig. 4:**
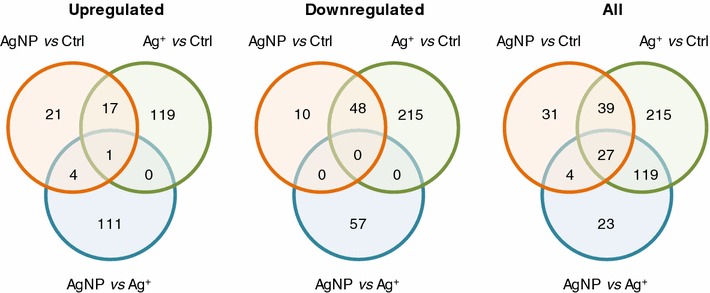
Venn Diagrams of upregulated, downregulated or all DEGS after exposure to AgNPs or Ag^+^ in comparison to the control, or compared with each other

The overlapping of DEGs between different treatment groups is plotted in the Venn diagrams shown in Fig. 4. Between the 43 genes upregulated by AgNP treatment and the 137 genes upregulated by Ag^+^ treatment, only 18 genes (17 plus 1) were shared by both groups. Likewise, for the downregulated genes, 58 for AgNP-treated group and 263 for Ag^+^-treated group, only 48 genes were common to both groups. These results suggest that there were substantial differences in the responses of cells to AgNPs and to Ag^+^ albeit some similarities exit.

### Functional annotation of differentially expressed genes

**Fig. 5:**
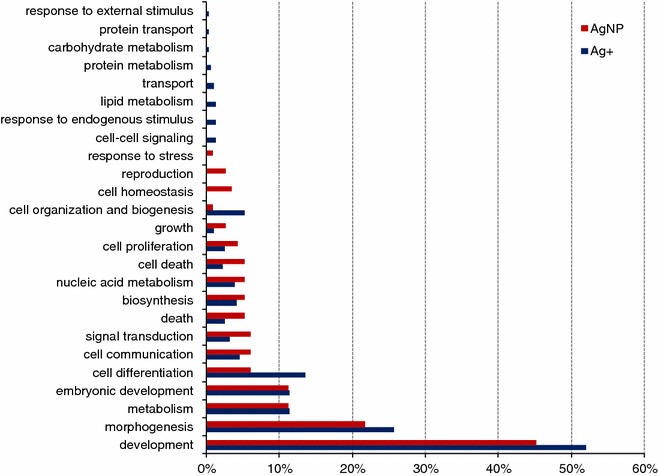
Distribution of enriched GO terms in various functional classes for the DEGs regulated by AgNPs or Ag^+^. The *vertical axis* lists all the classes generated by GO slim. The *horizontal axis* indicates the number of GO terms in each class as a percentage of the total number of unique GO terms enriched by the DEGs

To unravel the cellular functions affected by exposure to AgNPs and to Ag^+^, the DEGs were subjected to functional annotation using DAVID to find overrepresentations of gene ontology (GO) terms in the biological process (BP) category. The 101 genes regulated by AgNPs resulted in 120 GO terms (Additional file 6: Table S4). Using the CateGOrizer tool, these GO terms were grouped into 17 classes within the pre-defined set of parent/ancestor GO terms (Fig. 5). The 400 genes regulated by Ag^+^ led to 322 GO terms (Additional file 7: Table S5), which were further grouped into 22 GO classes (Fig. 5). Despite the large differences in the numbers of DEGs between AgNPs and Ag^+^, the functional classes enriched in these DEGs were strikingly similar between the two treatments. Fourteen classes were shared by AgNPs and Ag^+^; on top of the list were *development*, *morphogenesis*, *metabolism*, *embryonic development*, and *cell differentiation*. Some other classes were only regulated by Ag^+^ by not AgNPs, and vice versa. It is worthy to note that the class *response to stress* was enriched by AgNP treatment but not by Ag^+^.

Pathways impacted by AgNPs and by Ag^+^ were analyzed using IPA. Totally, 17 canonical pathways were affected by AgNPs and 27 by Ag^+^ (Table 3). These pathways were broadly classified into four major categories. In both cases, the majority of the pathways identified fell into two categories, embryonic development and metabolism. Nevertheless, three canonical pathways related to cancer were found for Ag^+^ but not for AgNPs. On the other hand, one pathway, *unfolded protein response*, was identified for AgNPs only.

**Table 3:** List of pathways impacted by AgNPs or Ag^+^ and DEGs involved in the pathways

Ingenuity Canonical Pathways	AgNPs		Ag^+^	
	p value	Molecules	p value	Molecules
Embryonic development				
Transcriptional regulatory network in embryonic stem cells	0.0005	HAND1, L1CAM, FOXC1	0.0000	HAND1, GATA6, L1CAM, EOMES, SKIL, HOXB1, FOXC1, GATA4
Human embryonic stem cell pluripotency	0.0178	SMAD7, SMAD6, BMP5	0.0074	BMP4, WNT3, BMP2, SMAD7, SMAD6, BMP5, FZD2
Mouse embryonic stem cell pluripotency			0.0002	LIFR, MYC, ID1, ID2, BMP4, T, ID3, FZD2
Role of NANOG in mammalian embryonic stem cell pluripotency			0.0001	LIFR, BMP4, T, WNT3, BMP2, GATA6, BMP5, FZD2, GATA4
Embryonic stem cell differentiation into cardiac lineages			0.0115	T, GATA4
Factors promoting cardiogenesis in vertebrates			0.0045	BMP4, WNT3, BMP2, BMP5, FZD2, GATA4
Cardiomyocyte differentiation via BMP receptors	0.0022	SMAD6, BMP5	0.0000	BMP4, BMP2, SMAD6, BMP5, GATA4
Regulation of the epithelial–mesenchymal transition pathway	0.0407	FOXC2, FGF14, PARD6B	0.0003	ETS1, FOXC2, ID2, FGF10, WNT3, FGF14, PARD6B, FGF3, NOTCH1, FZD2, FGF19
Hepatic fibrosis/hepatic stellate cell activation			0.0010	IGFBP4, COL4A1, FLT1, KLF6, SMAD7, LAMA1, BAMBI, IGFBP5, COL4A2, KDR
Axonal guidance signaling			0.0000	SLIT3, SHH, PAPPA, BMP4, WNT3, BMP2, UNC5B, L1CAM, HHIP, SLIT2, ROBO3, BMP5, NTN1, EFNB2, PRKAR2B, GLIS1, TUBB4A, FZD2, PTCH2, NRP1, UNC5C
BMP signaling pathway	0.0034	SMAD7, SMAD6, BMP5	0.0003	BMP4, PRKAR2B, BMP2, SMAD7, SMAD6, BMP5, CHRD
ERK5 signaling			0.0209	MYC, RPS6KA6, SGK1, CREB3L4
FGF signaling			0.0141	FGF10, FGF14, CREB3L4, FGF3, FGF19
Netrin signaling			0.0040	PRKAR2B, UNC5B, NTN1, UNC5C
Notch signaling			0.0004	DLL1, LFNG, DLL3, HES7, NOTCH1
RAR activation	0.0427	ALDH1A2, SMAD7, SMAD6	0.0148	PRKAR2B, CYP26A1, BMP2, ALDH1A2, SMAD7, SMAD6, ADCY8, RBP1
Sonic Hedgehog signaling			0.0001	SHH, PRKAR2B, GLIS1, HHIP, PTCH2
TGF-β signaling	0.0490	SMAD7, SMAD6		
eNOS signaling	0.0191	Hspa1b, HSPA1A/HSPA1B, KDR	0.0007	PRKAR2B, FLT1, HSPA1A/HSPA1B, PRKAA2, AQP8, ADCY8, KDR, LPAR3, NOSTRIN
VEGF family ligand-receptor interactions	0.0389	KDR, NRP1		
Metabolism				
Choline biosynthesis III	0.0490	HMOX1		
Corticotropin releasing hormone signaling			0.0389	SHH, PRKAR2B, CREB3L4, ADCY8, PTCH2
FXR/RXR activation			0.0200	TTR, APOB, VTN, VLDLR, MTTP, FGF19
Heme degradation	0.0166	HMOX1		
Histamine degradation	0.0490	ALDH1A2		
NAD biosynthesis II (from tryptophan)	0.0490	TDO2		
Retinoate biosynthesis I			0.0174	BMP2, ALDH1A2, RBP1
Serotonin degradation	0.0145	UGT2B28, ALDH1A2		
Sulfate activation for sulfonation			0.0331	PAPSS2
Tryptophan degradation to 2-amino-3-carboxymuconate semialdehyde	0.0288	TDO2		
Tyrosine biosynthesis IV			0.0490	PCBD1
Vitamin-C transport			0.0251	SLC23A1, GLRX
Xenobiotic metabolism signaling	0.0178	UGT2B28, HMOX1, ALDH1A2, CES2		
Stress response				
Unfolded protein response	0.0209	Hspa1b, HSPA1A/HSPA1B		
Cancer				
Molecular mechanisms of cancer			0.0013	SHH, Naip1 (includes others), BMP4, WNT3, BMP2, SMAD7, SMAD6, BMP5, MYC, CCND2, PRKAR2B, ADCY8, FZD2, NOTCH1, PTCH2
Basal cell carcinoma signaling			0.0000	SHH, BMP4, WNT3, GLIS1, BMP2, HHIP, BMP5, FZD2, PTCH2
Bladder cancer signaling			0.0155	MYC, FGF10, FGF14, FGF3, FGF19

**Fig. 6:**
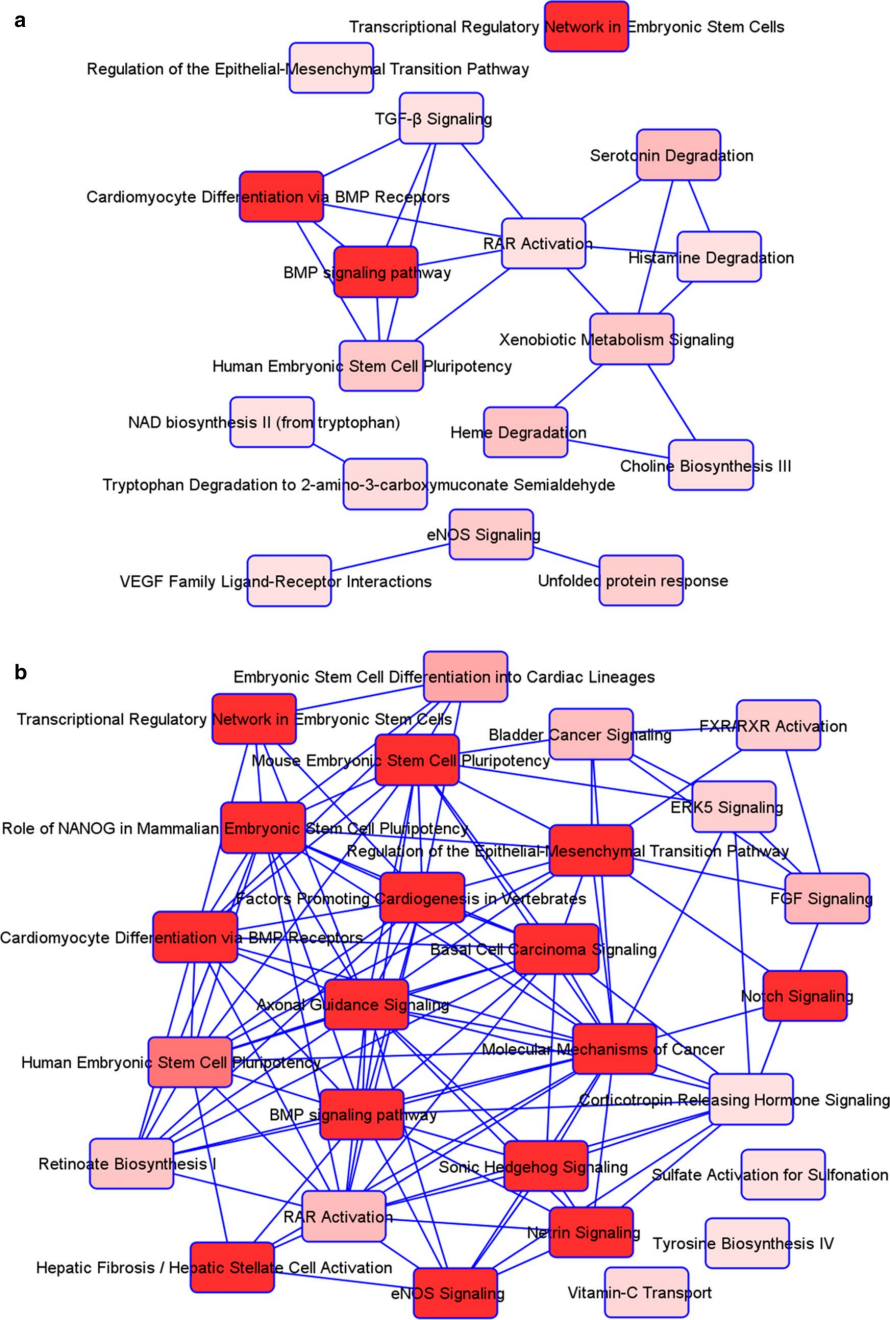
Pathway interaction networks for DEGs affected by **a** AgNPs and **b** Ag^+^. Each *rectangular box* represents a pathway affected by the DEGs with the name indicated. The *darkness* of the *red color* of each *box* represents the *p* value for enrichment of each pathway—the *darker the color*, the *lower* the *p* value. A *line* linking two *boxes* represents an interaction between two pathways. The length of the line is arbitrary

It was noted that for the common pathways shared by AgNPs and Ag^+^, there were always more genes involved in a particular pathway for Ag^+^ than for AgNPs, hence lower *p* values for the Ag^+^ pathways than their AgNP counterparts; and in almost all cases, the genes identified in AgNPs could be found in Ag^+^, suggesting more potent effect of Ag^+^ than AgNPs albeit of the same nature. This was also reflected by the pathway interaction networks shown in Fig. 6, where interactions among pathways impacted by Ag^+^ were much more intense that those by AgNPs.

### Oxidative stress and apoptosis regulated by AgNPs

**Fig. 7:**
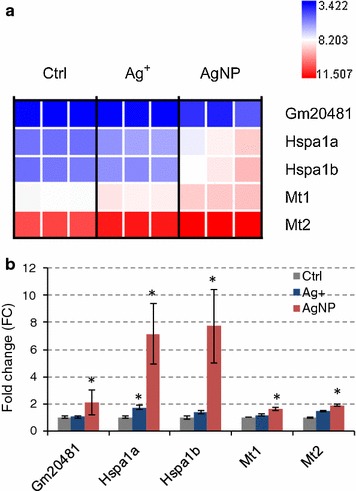
Upregulation of several members of the heat shock protein and the metallothionein families by AgNPs, but not Ag^+^. **a** Heat map showing the normalized expression intensity in the different treatment groups, which are in log2 scale and color coded as shown in the scheme at the *top right corner*. **b** *Bar graph* showing fold change (FC) of each member of the heat shock protein and the metallothionein families after exposure to AgNPs or to Ag^+^. The FC values are relative to the controls (of which the mean value was set to 1)

The *unfolded protein response* pathway identified for AgNPs (Table 3) prompted us to examine the expression of genes involved in oxidative stress, and several members of the heat shock protein and the metallothionein families were found significantly upregulated by AgNPs (Fig. 7). The increases in the expression of these genes in Ag^+^-exposed cells were minimal and not significant except for Hspa1a, of which the expression in Ag^+^-exposed cells was significantly (with *p* = 0.001086) higher than the control, but with |FC| marginally exceeded 1.5. These results suggest specific oxidative stress effect of AgNPs in ESCs.

**Fig. 8:**
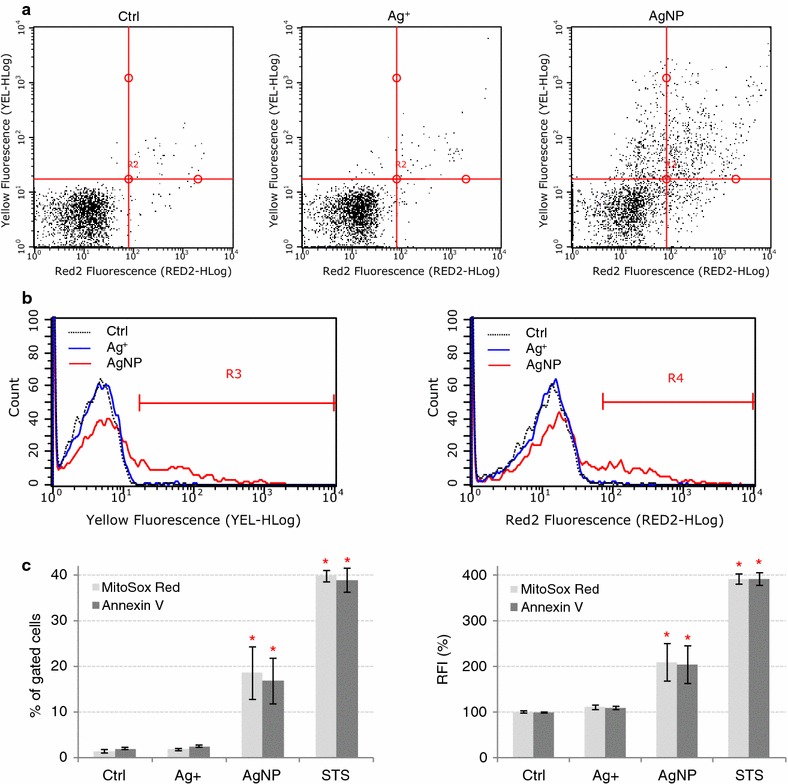
Flow cytometry results showing the effect of AgNPs and Ag^+^ on oxidative stress and apoptosis in mESCs. **a** Representative* dot plots* showing intensities of yellow fluorescence from MitoSox Red staining (oxidative stress) vs. red2 fluorescence from Annexin V binding (apoptosis) in control cells (Ctrl), and cells treated with Ag^+^ or AgNPs. **b** Representative histogram graphs showing fluorescence intensity distribution of yellow fluorescence from MitoSox Red staining (oxidative stress, *left*) and red2 fluorescence from Annexin V binding (apoptosis, *right*) in control cells (Ctrl), and cells treated with Ag^+^ or AgNPs. **c** *Bar graphs* showing the percentage of cells positive for MitoSox Red staining and Annexin V binding (gated cells in R3 and R4 of *B*, respectively; *left*), and mean relative fluorescence intensity (RFI; *right*) in total cell population of control cells (Ctrl), and cells treated with Ag^+^, AgNPs, or 1 µM staurosporine (STS). Values are expressed as percentage of the control and are mean ± SD of three independent experiments. *p < 0.05

To confirm these results, the effect of AgNPs on oxidative stress and downstream apoptosis were examined on the cellular level using flow cytometry (Fig. 8). Oxidative stress was assessed by mitochondrial superoxide generation which was detected by membrane permeant dye MitoSox Red. As shown in Fig. 8c, the percentage of cells positive for MitoSox Red staining (gated cells) increased significantly in AgNP treated cells (18.5%) as compared with control cells (1.4%). The mean intensity of yellow fluorescence (from MitoSox staining) of the entire cell population increased to 2.08-fold in cells treated with AgNPs in comparison with the control. As a comparison, treatment with 1 µM staurosporine, which has been reported to increases ROS production [34], resulted in 39.8% positive cells for MitoSox Red staining and 2.91-fold increase in mean fluorescence intensity compared with the control.

One of the hallmarks of apoptosis is the translocation of the membrane phospholipid phosphatidylserine to the external environment of the cells. Phosphatidylserine expression on the cell surface of apoptotic cells was assessed by binding of fluorescently labeled Annexin V in the current study. Similar to the results on oxidative stress as shown above, the percentage of cells positive for Annexin V binding (gated cells) increased significantly in AgNP treated cells (16.8%) as compared with control cells (1.9%), and the mean intensity of red2 fluorescence (from Annexin V binding) in cells treated with AgNPs was 2.04-fold as high as in control cells. For comparison, treatment with 1 µM staurosporine, a known apoptosis inducer [34], resulted in 38.9% positive cells for Annexin V binding and 2.91-fold increase in mean fluorescence intensity compared with the control.

Interestingly, cells treated with Ag^+^ did not show significant changes in either the percentage of gated cells or the mean fluorescence intensity, for both MitoSox Red staining and Annexin V binding, in comparison with the control cells (Fig. 8c).

## Discussion

The three-dimensional (3D) assembly of stem cells in the form of EB spheroids facilitates cellular interactions that promote morphogenesis, analogous to the multicellular, heterotypic tissue organization that accompanies embryogenesis [35]. The complex interactions between heterologous cell types result in the induction of differentiation of stem cells to derivatives of all three embryonic germ layers [36]. Therefore, ESC differentiation in EBs has been considered to faithfully recapitulate stages of early embryo development and is increasingly used as an in vitro model for developmental toxicity testing [13]. In addition, implementing toxicogenomics in ESC differentiation has been shown as a sensitive method for the detection of a variety of developmental toxicants [18]. In the present study, we characterized transcriptomic changes induced by citrate-coated 20 nm AgNPs during spontaneous differentiation of mESCs, and compared to those induced by Ag^+^ (silver acetate) under otherwise identical conditions. Despite the large differences in the numbers of DEGs, functional annotation and pathway analysis of the regulated genes revealed overall similarities between AgNPs and Ag^+^. Functions and pathways related to embryonic development and metabolism appeared on top of the lists for both treatments. Functional changes related to metabolism are thought to be necessary for cells to cope with the toxic insult of AgNPs and Ag^+^ [37]. Those related to embryonic development suggest that both AgNPs and Ag^+^ have the potential to cause developmental toxicities when in contact with the differentiating embryo. It has been reported that AgNPs induce distinct developmental defects in zebrafish embryos [38]. In a recent in vivo study, it was found that silver acetate exposure caused adverse effects on reproduction and postnatal development in rats [39].

Human exposure to nanoparticles may occur via inhalation, ingestion, dermal absorption or, in some cases, artificially induced via inoculation. Once systemically available, these nanoparticles appear capable of spreading to most organ systems and may even cross biological barriers. Several studies demonstrated that certain nanoparticles can penetrate the placenta barrier, reach the fetus, and evoke embryotoxic effects [40]. Since the application of AgNPs is expected to further increase in the future, long term exposure and potential accumulation of AgNPs in the human body may result. The results presented here point to the potential of AgNPs to cause developmental toxicity once these particles migrate into the uterus, cross the placenta and reach the embryo.

Proper physicochemical characterization of nanoparticles should be performed in the relevant dispersion medium prior to conducting toxicity studies [41, 42]. This is because complex interactions exists between a particle and its surrounding microenvironment, including attractive or repulsive forces between particles, and between particles and biological substances in the dispersion medium such as salts (ions) and proteins. These factors affect both the hydrodynamic size and the surface charge of the nanoparticles, and can impact on their agglomeration status, which may in turn affect the extent of toxicity. It has been considered that larger agglomerates of nanoparticles are less toxic than monodispersed particles or smaller aggregates [41]. The DLS and UV–Vis data suggest there was a dynamic interplay between the AgNPs and the culture medium, starting immediately upon contact and lasting throughout the duration of the exposure, leading to increased agglomeration of the AgNPs. Aggregation of nanoparticles in cell culture media has been reported previously by others [19, 37, 40]. However, it has to be noted that there are significant limitations to the techniques used for the characterization, which complicates the interpretation of the data. For both DLS and UV–Vis, characteristic peaks for AgNPs overlaps with those of the medium. Moreover, it is well known that DLS is a weight-averaged measurement biased towards larger particle sizes and especially towards agglomerates or aggregates. Thus, although the observed DLS curve showed an average hydrodynamic size of ~78 nm, the system may actually contain a very high number of smaller particles that are bioavailable and can interact with the ESCs. From another point of view, it should be pointed out that the agglomeration of AgNPs found here does not diminish, but rather adds to, the significance of the current study. Since the culture medium for ESCs used here was similar to body fluid in composition, it is likely that AgNPs entering the human body would agglomerate to some extent. In this sense, the results found in the current study are meaningful for real life situations. Since unagglomerated (monodispersed) form of nanoparticles would be more toxic than their agglomerated form, the results reported here signify the importance of nanoparticle regulation in consumer products.

It was interesting to note that in the EB state ESCs were in general more resistant to AgNP and Ag^+^ cytotoxicity than in adherent culture (Fig. 2). At 5.0 μg/ml, Ag^+^ caused nearly complete cell death (97%) in adherent culture, while >80% cells survived in EBs. For AgNPs, cell viability at 5.0 μg/ml was 68% in adherent culture and 87% in EB state. At 50 µg/ml, 73 and 34% cells remained alive after exposure to AgNPs and Ag^+^ respectively in EB culture. In comparison, in adherent culture only 43% cells survived AgNP exposure, and <2% cells survived after exposing to Ag^+^. These results could be explained by the fact that in adherent culture, cells formed a monolayer whereby AgNPs or Ag^+^ diffused freely throughout the medium, and thereby reached equilibrium where all cells were equally exposed to the same concentrations of AgNPs or Ag^+^. In contrast, in 3D aggregates of EBs, a concentration gradient of exogenous or endogenous factors is established between the surrounding culture environment and the interior of the spheroids [35]. Therefore, concentration of Ag^+^ in the interior of the EBs would be lower than that of exterior environment. The concentration is inversely related to EB size, with decreasing concentration from the surface toward the center of aggregates [35]. Due to the high cell packing density (Additional file 2: Figure S2), cells in the center of the EBs may be completely shielded and not exposed to Ag^+^ at all. For AgNPs, the concentration in the interior of the EBs would be ever lower than Ag^+^ due to more severe steric hindrance imposed on the AgNPs as a result of their larger sizes, especially if agglomeration occurred.

It was also intriguing to note that in adherent cultures, low concentrations of Ag^+^ (up to 0.5 μg/ml) increased cell viability almost 20% relative to the unexposed control, suggesting accelerated mESC proliferation and/or differentiation (Fig. 2a). Similar stimulating effects were also observed for AgNPs, albeit not statistically significant (Fig. 2a). Such an hormesis effect was also reported previously for AgNPs in HepG2 cells [24], and for silica nanoparticles in D3 mESCs [40], and could be explained as an adaptive response of cells to low levels of potentially toxic agents [40].

The toxicity of AgNPs has been demonstrated both in vitro and in vivo [43]. However, whether the observed toxicity is due to Ag^+^ released from the AgNPs or related to the special properties of nanosized particles is not entirely clear, and is often a topic of rigorous debate. This is partly due to the fact that the release of Ag^+^ from AgNPs is a dynamic process and is affected by many factors such as temperature, surface chemistry, and stabilizing agent [44]. In order to unravel the mechanism of toxicity, the effect of AgNPs on the gene expression in ESCs was studied here at a relatively low toxicity concentration (~EC10) to avoid cellular processes in necrotic or apoptotic cells overwhelming and leading to misinterpretation of the data. As comparison, two concentrations of Ag^+^ were used, one approximates the maximum Ag^+^ released from 5.0 μg/ml AgNPs after 24 h incubation in the medium (1.0 μg/ml; Additional file 1: Figure S1), the other was the equivalent mass concentration of silver in 5.0 μg/ml AgNPs (i.e., 5.0 μg/ml). At 1.0 μg/ml, Ag^+^ only induced 47 DEGs (data not shown), compared to 101 DEGs regulated by 5.0 μg/ml AgNPs. This suggests that AgNP toxicity was not entirely due to Ag^+^ released from the AgNPs. In the present study, we only measured Ag^+^ release from AgNPs in the medium (without cells). AgNPs may also release Ag^+^ inside the cell via a “Trojan-horse” mechanism, where the particles enter cells and are then ionized within the cell [45]. The purpose of the present study was to evaluate the effect of AgNPs and Ag^+^ on the cellular transcriptome of ESCs, and therefore more systematic studies are needed in order to completely disentangle the effects of AgNPs from those of released Ag^+^. In addition, a cellular uptake study of the AgNPs may help to confirm that the transcriptomic changes seen here were indeed caused by ingested AgNPs.

More importantly, microarray data analysis showed that several stress response proteins, including members of the heat shock proteins (HSPs) and the metallothionein (MT) families, were upregulated by AgNPs but not Ag^+^. As the name suggests, HSPs are a group of proteins induced by heat shock, or hyperthermia. Their expression has also been found to increase when cells are exposed to an array of other stresses, including heavy metals and oxygen radicals [46], where they play a role in maintaining the correct folding of proteins by preventing protein aggregation or facilitating selective degradation of misfolded or denatured proteins [47]. MTs are a family of cysteine-rich proteins with several isoforms [48]. MTs have the capacity to bind heavy metals, both physiological and xenobiotic, through the thiol group of their cysteine residues [49]. It has been suggested that MTs not only are involved in the regulation of physiological metals and protect cells from metal toxicity, but also provide protection against oxidative stress [50]. The cysteines of MTs have been shown to bind oxidant radicals like superoxide and hydroxyl radicals [51], and MT expression has been implicated as a transient response to many forms of stress or injury [50]. Several previous studies [23, 24, 37, 52, 53] reported upregulation of HSPs and MTs in somatic cells following exposure to AgNPs. The upregulation of these proteins found in the current study suggests that AgNP exposure also induced cellular stresses and elicited cellular protective responses in ESCs. It was intriguing to note that the same concentration of Ag^+^ did not induce such stress responses in the ESCs. The reason for this is not well understood but could be partly explained by the so-called Trojan horse theory. The plasma membrane functions to some degree as a natural barrier for metal ions, thereby protecting the cells from damage. However, nanoparticles circumvent this barrier when they are taken up by the cells via endocytic pathways, leading to the release of metal ions within the cells as a result of lysosome rupture, and subsequently generate free radicals within the cells [23]. Similar findings were reported previously [23, 24].

**Fig. 9:**
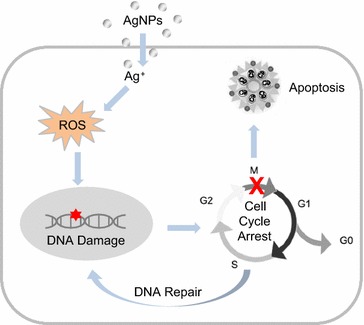
Schematic representation of molecular mechanisms of AgNP toxicity in ESCs, which are similar to those previously reported for somatic cells. AgNPs enter ESCs via endocytosis, release Ag^+^ within the cell after lysosome rupture, and subsequently generate ROS. The elevated ROS may lead to DNA damage and cause the cell into cycle arrest in order to repair damaged DNA. Cells failed to do so will eventually undergo apoptosis

It has been reported that in several somatic cell types, augmented oxidative stress induced by AgNPs, through the generation of reactive oxygen species (ROS), may further lead to DNA damage and chromosomal aberrations [19, 53, 54]. Cells with damaged DNA will accumulate in the G2/M phase allowing the cells extra time to repair of damaged DNA, causing arrest in cell cycle progression [23, 54]. Cells with massive or irreversible DNA damage will not be able to repair the DNA effectively and undergo apoptosis at a later stage [19, 53, 54]. The results presented here indicate that this scenario may also hold true for ESCs. Flow cytometry results (Fig. 8) indicated that both oxidative stress and apoptosis increased significantly after treatment with AgNPs. Based on these results, the molecular mechanisms of ESC cellular responses against AgNPs are therefore speculated as following (Fig. 9): AgNPs enter the cell via endocytosis, release Ag^+^ within the cell after lysosome rupture, and subsequently generate ROS. The increased oxidative stress further leads to DNA damage, causing cell cycle arrest at the G2/M phase in order to repair the damaged DNA. Cells unable to repair the DNA damages will eventually undergo apoptosis.

Kawata et al. [24] found a number of checkpoint related genes (BIRC5, BUB1B, CCNA2, CDC25B, CDC20, and CKS2) increased expression in HepG2 cells after exposure to AgNPs. However, despite the effect of AgNPs on oxidative stress and apoptosis revealed at the protein level by flow cytometry, significant induction of genes associated with the cellular processes downstream of oxidative stress (DNA damage, cell cycle arrest, and apoptosis) was not observed at the mRNA level by the microarray study. This is probably because of the ephemeral nature of many gene expression changes. The correlation between mRNA and protein abundances in the cell has been reported to be notoriously poor [55]. One of the reasons for the poor correlation is that there is a temporal difference in cells responding to environment perturbations at the mRNA level and at the protein level [56]. There may have been detectable gene expression changes at the mRNA level; however, by the time when these changes are translated into the protein level, the altered mRNA expression of these genes may have already recovered to their normal level.

DNA damage by ROS production induced by AgNPs may further activate the p53 pathway [19], which plays important roles in carcinogenesis and tumor progressing. Therefore, AgNPs have the potential to cause carcinogenicity [24]. However, pathways related to cancer were not identified in cells exposed to AgNPs. On the contrary, three pathways were found in cells exposed to Ag^+^. The reason for this is not clear. However, it cannot be excluded that AgNPs would affect cancer pathways at higher concentrations than that used in the current study (5.0 µg/ml).

## Conclusions

In this study, we characterized transcriptomic changes induced by AgNPs during spontaneous differentiation of mouse ESCs, and compared to those induced by Ag^+^. Overall, cellular responses to AgNPs and Ag^+^ in ESCs were substantially similar. In both cases, most of the functions and pathways impacted are related to embryonic development and metabolism, suggesting that both AgNPs and Ag^+^ have the potential to alter, reversibly or irreversibly, developmental pathways. However, specific effects on oxidative stress and apoptosis were observed for AgNPs. Taken together, our results indicate that the widespread application of AgNPs and silver-containing products may be a health concern. Long term exposure of humans to these products could potentially result in accumulation in the body and subsequently induce acute or chronic toxicity. Once systemically available, AgNPs has the potential to reach the embryo thus causing developmental toxicity. However, since information on whether these particles are able to transfer from the mother to the fetus across the placenta is currently lacking, the results of the current study have to be interpreted with caution until in vivo data becomes available.

## Additional files

**Additional file 1: Figure S1.** Release of Ag^+^ from AgNPs into culture medium after 24 h incubation at 37 °C. (A) Ag^+^ released into the supernatant of the medium before (0 h) and after (24 h) incubation. (B) Total Ag^+^ in the AgNP suspension before (0 h) and after (24 h) incubation.

**Additional file 2: Figure S2.** Morphological changes of the differentiating EBs after exposure for 24 h to varying concentrations of AgNPs (A) or Ag^+^ (B). The concentrations of AgNPs or Ag^+^ used for the exposure (in µg/ml) are indicated by the numbers at the top left corner of each image.

**Additional file 3: Table S1.** List of 101 DEGs regulated by AgNPs. Gene expression profiles of AgNP-exposed samples were compared with the controls using one-way analysis of variance (ANOVA) based on the Welch *t*-test, and DEGs were selected by |FC| ≥ 1.5 and *p* ≤ 0.05.

**Additional file 4: Table S2.** List of 400 DEGs regulated by Ag^+^. Gene expression profiles of Ag^+^-exposed samples were compared with the controls using one-way analysis of variance (ANOVA) based on the Welch *t* test, and DEGs were selected by |FC| ≥ 1.5 and *p* ≤ 0.05.

**Additional file 5: Table S3.** List of 173 DEGs between AgNP- and Ag^+^- treated cells. Gene expression profiles of AgNP-exposed samples were compared with Ag^+^-exposed samples using one-way analysis of variance (ANOVA) based on the Welch *t* test, and DEGs were selected by |FC| ≥ 1.5 and *p* ≤ 0.05.

**Additional file 6: Table S4.** Lists of 120 GO terms in the biological process (BP) category enriched from the 101 genes regulated by AgNPs. DAVID was used for the analysis. The *Mus musculus* (mouse) whole genome was used as background. Statistical enrichment was determined through a modified Fisher’s exact test (*p* < 0.1) and count threshold >2 (default settings in DAVID).

**Additional file 7: Table S5.** Lists of 322 GO terms in the biological process (BP) category enriched from the 400 genes regulated by Ag^+^. DAVID was used for the analysis. The *Mus musculus* (mouse) whole genome was used as background. Statistical enrichment was determined through a modified Fisher’s exact test (*p* < 0.1) and count threshold >2 (default settings in DAVID).

## Abbreviations

3D: three-dimensional
AGCC: Affymetrix GeneChip Command Console
Ag^+^: silver ion
AgNO_3_: silver nitrate
AgNP: silver nanoparticle
ANOVA: analysis of variance
BMP-4: bone morphogenic protein 4
BP: biological process
DAVID: Database for Annotation, Visualization, and Integrated Discovery
DEG: differentially expressed gene
DI: deionized
DLS: dynamic light scattering
EB: embryoid body
ECVAM: European Committee for the Validation of Alternative Methods
ESC: embryonic stem cell
EST: embryonic stem cell test
FC: fold change
GEO: Gene Expression Omnibus
GO: gene ontology
GSK3b-I: glycogen synthase kinase-3b inhibitor
HCA: hierarchical cluster analysis
HSP: heat shock protein
ICP-MS: inductively coupled plasma mass spectroscopy
IPA: Ingenuity Pathway Analysis
LIF: leukemia inhibitory factor
mESC: mouse embryonic stem cell
MT: metallothionein
PBS: phosphate buffered saline
PCA: principal component analysis
PDI: polydispersity index
RMA: robust multi-array average
ROS: reactive oxygen species
STS: staurosporine
TAC: Transcriptome Analysis Console
TEM: transmission electron microscopy
UV-vis: ultraviolet-visible

## References

[1] The Woodrow Wilson International Center. Consumer products inventory. http://www.nanotechproject.org/cpi/. Accessed Apr 2016.

[2] Henig RM. Our silver-coated future. 2007. http://archive.onearth.org/article/our-silver-coated-future. Accessed Feb 2015.

[3] Lara HH, Garza-Treviño EN, Ixtepan-Turrent L, Singh DK (2011). Silver nanoparticles are broad-spectrum bactericidal and virucidal compounds. J Nanobiotechnol.

[4] You C, Han C, Wang X, Zheng Y, Li Q, Hu X, Sun H (2012). The progress of silver nanoparticles in the antibacterial mechanism, clinical application and cytotoxicity. Mol Biol Rep.

[5] Scientific Committee on Emerging and Newly Identified Health Risks (SCENIHR). Opinion on nanosilver: safety, health and environmental effects and role in antimicrobial resistance, 2014. http://ec.europa.eu/health/scientific_committees/emerging/docs/scenihr_o_039.pdf. Accessed Apr 2016.

[6] Bartłomiejczyk T, Lankoff A, Kruszewski M, Szumiel I (2013). Silver nanoparticles—allies or adversaries?. Ann Agric Environ Med.

[7] Zhang T, Wang L, Chen Q, Chen C (2014). Cytotoxic potential of silver nanoparticles. Yonsei Med J.

[8] Kim YS, Kim JS, Cho HS, Rha DS, Kim JM, Park JD, Choi BS, Lim R, Chang HK, Chung YH, Kwon IH, Jeong J, Han BS, Yu IJ (2008). Twenty-eight-day oral toxicity, genotoxicity, and gender-related tissue distribution of silver nanoparticles in Sprague–Dawley rats. Inhal Toxicol.

[9] Kim YS, Song MY, Park JD, Song KS, Ryu HR, Chung YH, Chang HK, Lee JH, Oh KH, Kelman BJ, Hwang IK, Yu IJ (2010). Subchronic oral toxicity of silver nanoparticles. Part Fibre Toxicol.

[10] Tang J, Xiong L, Wang S, Wang J, Liu L, Li J, Yuan F, Xi T (2009). Distribution, translocation and accumulation of silver nanoparticles in rats. J Nanosci Nanotechnol.

[11] Tang J, Xiong L, Zhou G, Wang S, Wang J, Liu L, Li J, Yuan F, Lu S, Wan Z, Chou L, Xi T (2010). Silver nanoparticles crossing through and distribution in the blood-brain barrier in vitro. J Nanosci Nanotechnol.

[12] Doran KS, Banerjee A, Disson O, Lecuit M (2013). Concepts and mechanisms: crossing host barriers. Cold Spring Harb Perspect Med.

[13] Tandon S, Jyoti S (2012). Embryonic stem cells: an alternative approach to developmental toxicity testing. J Pharm Bioallied Sci.

[14] Heuer J, Bremer S, Pohl I, Spielmann H (1993). Development of an in vitro embryotoxicity test using murine embryonic stem cell cultures. Toxicol In Vitro.

[15] Genschow E, Spielmann H, Scholz G, Pohl I, Seiler A, Clemann N, Bremer S, Becker K (2004). Validation of the embryonic stem cell test in the international ECVAM validation study on three in vitro embryotoxicity tests. Altern Lab Anim.

[16] Paquette JA, Kumpf SW, Streck RD, Thomson JJ, Chapin RE, Stedman DB (2008). Assessment of the embryonic stem cell test and application and use in the pharmaceutical industry. Birth Defects Res B Dev Reprod Toxicol.

[17] van Dartel DA, Piersma AH (2011). The embryonic stem cell test combined with toxicogenomics as an alternative testing model for the assessment of developmental toxicity. Reprod Toxicol.

[18] Gao X, Yourick JJ, Sprando RL (2014). Transcriptomic characterization of C57BL/6 mouse embryonic stem cell differentiation and its modulation by developmental toxicants. PLoS ONE.

[19] Ahamed M, Karns M, Goodson M, Rowe J, Hussain SM, Schlager JJ, Hong Y (2008). DNA damage response to different surface chemistry of silver nanoparticles in mammalian cells. Toxicol Appl Pharmacol.

[20] Park MV, Neigh AM, Vermeulen JP, de la Fonteyne LJ, Verharen HW, Briedé JJ, van Loveren H, de Jong WH (2011). The effect of particle size on the cytotoxicity, inflammation, developmental toxicity and genotoxicity of silver nanoparticles. Biomaterials.

[21] Samberg ME, Loboa EG, Oldenburg SJ, Monteiro-Riviere NA (2012). Silver nanoparticles do not influence stem cell differentiation but cause minimal toxicity. Nanomedicine.

[22] Bouwmeester H, Poortman J, Peters RJ, Wijma E, Kramer E, Makama S, Puspitaninganindita K, Marvin HJ, Peijnenburg AA, Hendriksen PJ (2011). Characterization of translocation of silver nanoparticles and effects on whole-genome gene expression using an in vitro intestinal epithelium coculture model. ACS Nano.

[23] Foldbjerg R, Irving ES, Hayashi Y, Sutherland DS, Thorsen K, Autrup H, Beer C (2012). Global gene expression profiling of human lung epithelial cells after exposure to nanosilver. Toxicol Sci.

[24] Kawata K, Osawa M, Okabe S (2009). In vitro toxicity of silver nanoparticles at noncytotoxic doses to HepG2 human hepatoma cells. Environ Sci Technol.

[25] De Smedt A, Steemans M, De Boeck M, Peters AK, van der Leede BJ, Van Goethem F, Lampo A, Vanparys P (2008). Optimisation of the cell cultivation methods in the embryonic stem cell test results in an increased differentiation potential of the cells into strong beating myocard cells. Toxicol In Vitro.

[26] Irizarry RA, Hobbs B, Collin F, Beazer-Barclay YD, Antonellis KJ, Scherf U, Speed TP (2003). Exploration, normalization, and summaries of high density oligonucleotide array probe level data. Biostatistics.

[27] Ringnér M (2008). What is principal component analysis?. Nat Biotechnol.

[28] Eisen MB, Spellman PT, Brown PO, Botstein D (1998). Cluster analysis and display of genome-wide expression patterns. Proc Natl Acad Sci USA.

[29] Tong W, Cao X, Harris S, Sun H, Fang H, Fuscoe J, Harris A, Hong H, Xie Q, Perkins R, Shi L, Casciano D (2003). Arraytrack-supporting toxicogenomic research at the U.S. Food and Drug Administration National Center for Toxicological Research. Environ Health Perspect.

[30] Tong W, Harris S, Cao X, Fang H, Shi L, Sun H, Fuscoe J, Harris A, Hong H, Xie Q, Perkins R, Casciano D (2004). Development of public toxicogenomics software for microarray data management and analysis. Mutat Res.

[31] Dennis G, Sherman BT, Hosack DA, Yang J, Gao W, Lane HC, Lempicki RA (2003). DAVID: database for annotation, visualization, and integrated discovery. Genome Biol.

[32] da Huang W, Sherman BT, Lempicki RA (2009). Systematic and integrative analysis of large gene lists using DAVID bioinformatics resources. Nat Protoc.

[33] Hu ZL, Bao J, Reecy JM (2008). CateGOrizer: a web-based program to batch analyze gene ontology classification categories. Online J Bioinform.

[34] Pong K, Doctrow SR, Huffman K, Adinolfi CA, Baudry M (2001). Attenuation of staurosporine-induced apoptosis, oxidative stress, and mitochondrial dysfunction by synthetic superoxide dismutase and catalase mimetics, in cultured cortical neurons. Exp Neurol.

[35] Kinney MA, Hookway TA, Wang Y, McDevitt TC (2014). Engineering three-dimensional stem cell morphogenesis for the development of tissue models and scalable regenerative therapeutics. Ann Biomed Eng.

[36] Martin GR, Wiley LM, Damjanov I (1977). The development of cystic embryoid bodies in vitro from clonal teratocarcinoma stem cells. Dev Biol.

[37] Xu L, Takemura T, Xu M, Hanagata N (2011). Toxicity of silver nanoparticles as assessed by global gene expression analysis. Mater Express.

[38] Asharani PV, Lian WuY, Gong Z, Valiyaveettil S (2008). Toxicity of silver nanoparticles in zebrafish models. Nanotechnology.

[39] Sprando RL, Black T, Keltner Z, Olejnik N, Ferguson M (2016). Silver acetate exposure: effects on reproduction and post natal development. Food Chem Toxicol.

[40] Park MV, Annema W, Salvati A, Lesniak A, Elsaesser A, Barnes C, McKerr G, Howard CV, Lynch I, Dawson KA, Piersma AH, de Jong WH (2009). In vitro developmental toxicity test detects inhibition of stem cell differentiation by silica nanoparticles. Toxicol Appl Pharmacol.

[41] Boverhof DR, David RM (2010). Nanomaterial characterization: considerations and needs for hazard assessment and safety evaluation. Anal Bioanal Chem.

[42] Warheit DB (2008). How meaningful are the results of nanotoxicity studies in the absence of adequate material characterization?. Toxicol Sci.

[43] Ge L, Li Q, Wang M, Ouyang J, Li X, Xing MM (2014). Nanosilver particles in medical applications: synthesis, performance, and toxicity. Int J Nanomed.

[44] Kittler S, Greulich C, Diendorf J, Koller M, Epple M (2010). Toxicity of silver nanoparticles increases during storage because of slow dissolution under release of silver ions. Chem Mater.

[45] Park EJ, Yi J, Kim Y, Choi K, Park K (2010). Silver nanoparticles induce cytotoxicity by a Trojan-horse type mechanism. Toxicol In Vitro.

[46] De Maio A (1999). Heat shock proteins: facts, thoughts, and dreams. Shock.

[47] Gupta SC, Sharma A, Mishra M, Mishra RK, Chowdhuri DK (2010). Heat shock proteins in toxicology: how close and how far?. Life Sci.

[48] Hunziker PE, Kägi JH (1985). Isolation and characterization of six human hepatic isometallothioneins. Biochem J.

[49] Davis SR, Cousins RJ (2000). Metallothionein expression in animals: a physiological perspective on function. J Nutr.

[50] Theocharis SE, Margeli AP, Koutselinis A (2003). Metallothionein: a multifunctional protein from toxicity to cancer. Int J Biol Markers.

[51] Ruttkay-Nedecky B, Nejdl L, Gumulec J, Zitka O, Masarik M, Eckschlager T, Stiborova M, Adam V, Kizek R (2013). The role of metallothionein in oxidative stress. Int J Mol Sci.

[52] Sahu SC, Zheng J, Yourick JJ, Sprando RL, Gao X (2015). Toxicogenomic responses of human liver HepG2 cells to silver nanoparticles. J Appl Toxicol.

[53] Xu L, Li X, Takemura T, Hanagata N, Wu G, Chou LL (2012). Genotoxicity and molecular response of silver nanoparticle (NP)-based hydrogel. J Nanobiotechnol.

[54] AshaRani PV, Low Kah Mun G, Hande MP, Valiyaveettil S (2009). Cytotoxicity and genotoxicity of silver nanoparticles in human cells. ACS Nano.

[55] Maier T, Güell M, Serrano L (2009). Correlation of mRNA and protein in complex biological samples. FEBS Lett.

[56] Chechik G, Koller D (2009). Timing of gene expression responses to environmental changes. J Comput Biol.

