# Supplementary material for: Toxicity of nano- and ionic silver to embryonic stem cells: a comparative toxicogenomic study
Source: J Nanobiotechnology. 2017 Apr 11;15:31. doi: 10.1186/s12951-017-0265-6 (PMC5387260; doi:10.1186/s12951-017-0265-6)
Supplement: Supplementary file 1 — Additional file 1: Figure S1. Release of Ag+ from AgNPs into culture medium after 24 h incubation at 37 °C. (A) Ag+ released into the supernatant of the medium before (0 h) and after (24 h) incubation. (B) Total Ag+ in the AgNP suspension before (0 h) and after (24 h) incubation. [file 12951_2017_265_MOESM1_ESM.pptx]

## Slide 1
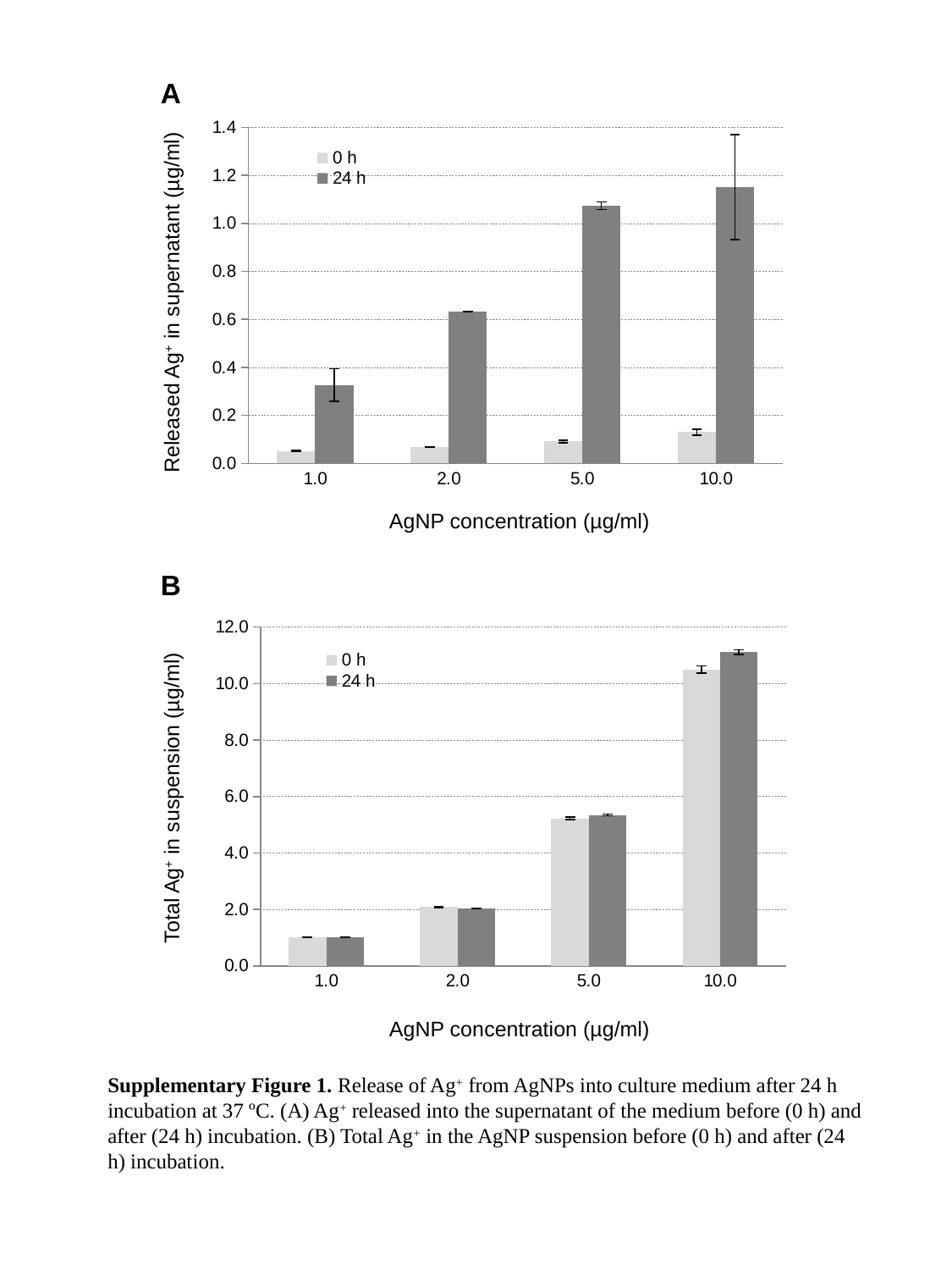

A
### Chart
| Category | | |
|---|---|---|
| 1 | 0.05220384820502869 | 0.3264084575373949 |
| 2 | 0.06893197689378372 | 0.6311683953605832 |
| 5 | 0.09130980807068712 | 1.0731769170078014 |
| 10 | 0.12998385800778875 | 1.1514320672560114 |Released Ag+ in supernatant (µg/ml)
AgNP concentration (µg/ml)
B
### Chart
| Category | | |
|---|---|---|
| 1 | 1.0013334391837214 | 1.0175012396627636 |
| 2 | 2.0830301942678298 | 2.0296620989824916 |
| 5 | 5.2245252322190625 | 5.342381668531764 |
| 10 | 10.494633385727754 | 11.108917067752465 |Total Ag+ in suspension (µg/ml)
AgNP concentration (µg/ml)
Supplementary Figure 1. Release of Ag+ from AgNPs into culture medium after 24 h incubation at 37 ºC. (A) Ag+ released into the supernatant of the medium before (0 h) and after (24 h) incubation. (B) Total Ag+ in the AgNP suspension before (0 h) and after (24 h) incubation.
