# Supplementary material for: Toxicity of nano- and ionic silver to embryonic stem cells: a comparative toxicogenomic study
Source: J Nanobiotechnology. 2017 Apr 11;15:31. doi: 10.1186/s12951-017-0265-6 (PMC5387260; doi:10.1186/s12951-017-0265-6)
Supplement: Supplementary file 2 — Additional file 2: Figure S2. Morphological changes of the differentiating EBs after exposure for 24 h to varying concentrations of AgNPs (A) or Ag+ (B). The concentrations of AgNPs or Ag+ used for the exposure (in µg/ml) are indicated by the numbers at the top left corner of each image. [file 12951_2017_265_MOESM2_ESM.pptx]

## Slide 1
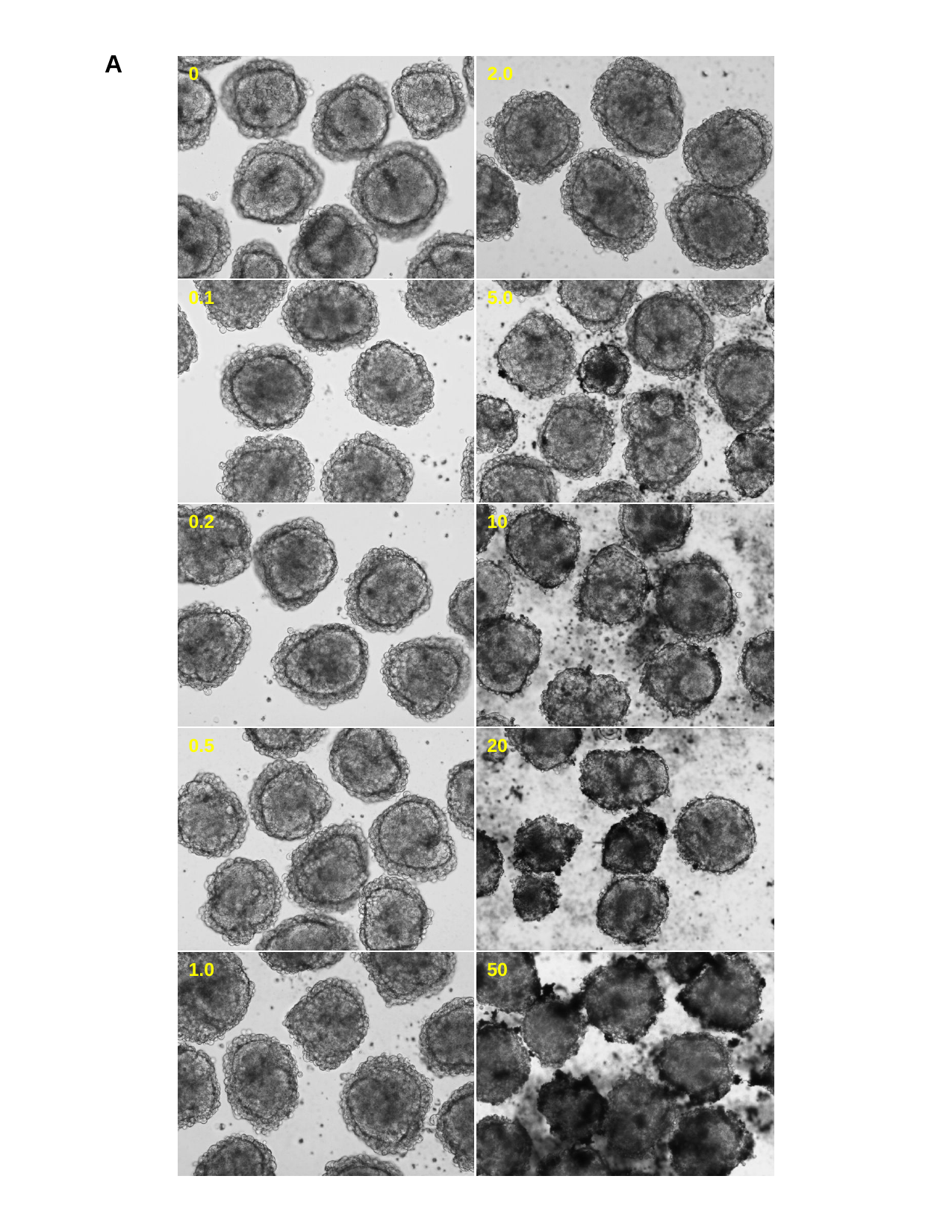

A
0
2.0
0.1
5.0
0.2
10
0.5
20
1.0
50

## Slide 2
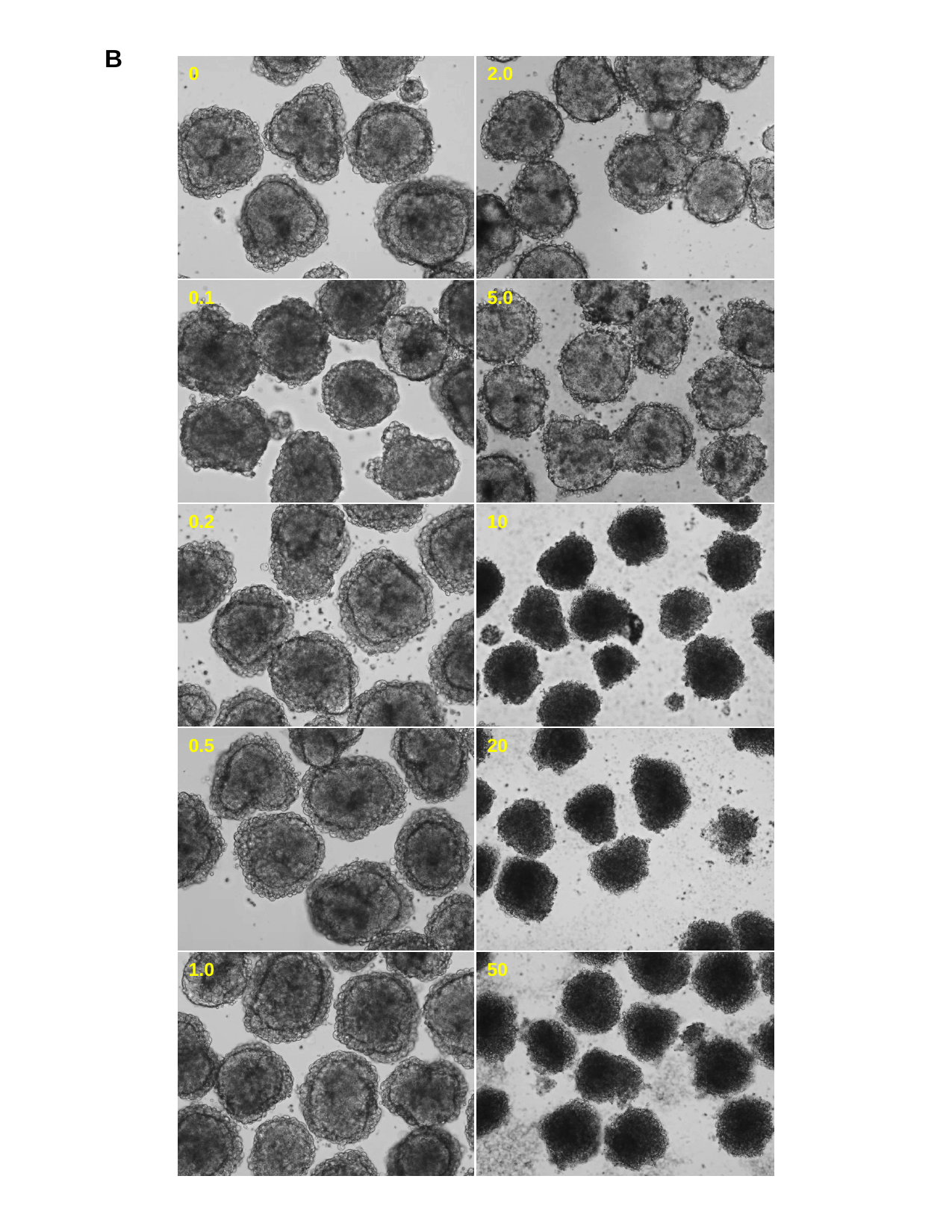

B
0
2.0
0.1
5.0
0.2
10
0.5
20
1.0
50

## Slide 3
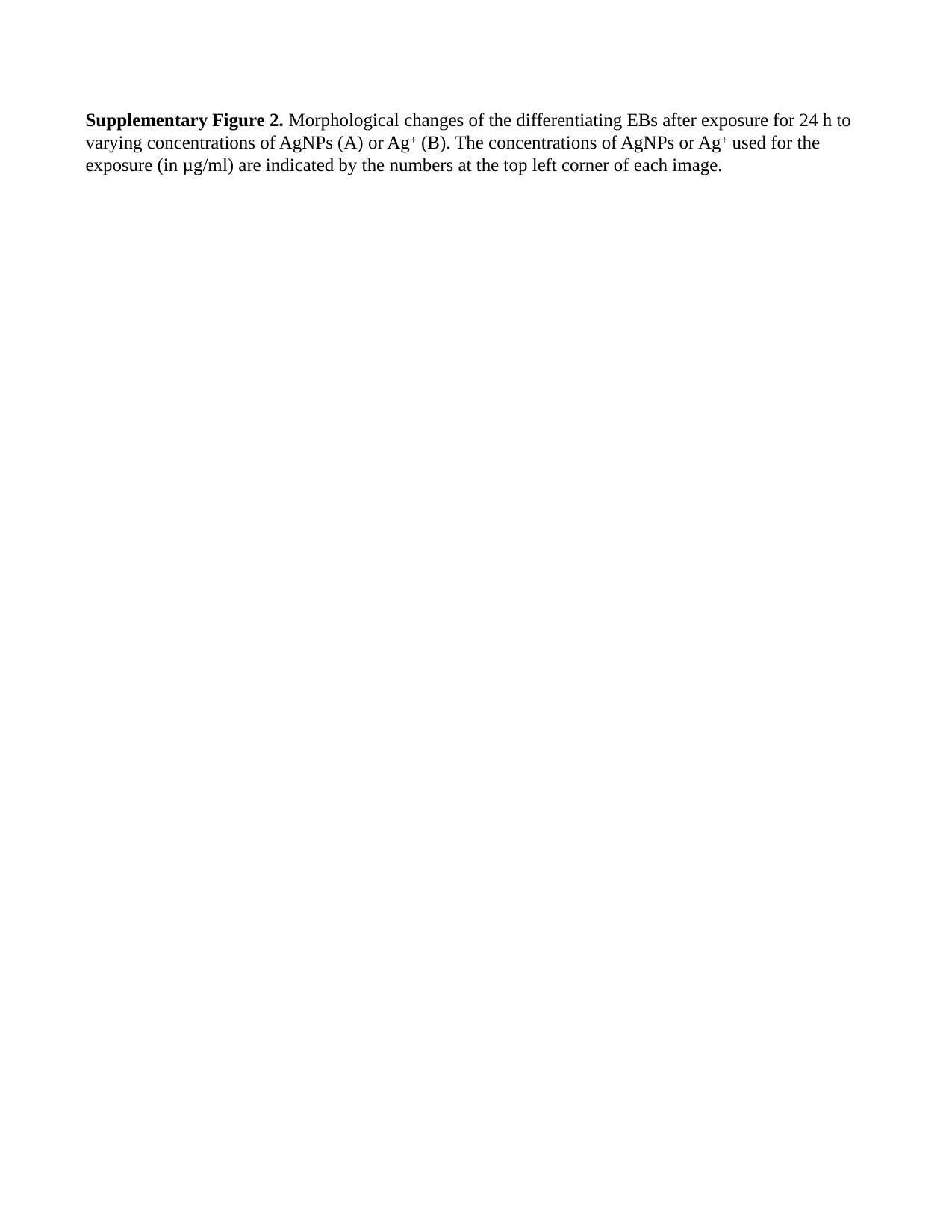

Supplementary Figure 2. Morphological changes of the differentiating EBs after exposure for 24 h to varying concentrations of AgNPs (A) or Ag+ (B). The concentrations of AgNPs or Ag+ used for the exposure (in µg/ml) are indicated by the numbers at the top left corner of each image.
